# Vision-Based Attentiveness Determination Using Scalable HMM Based on Relevance Theory

**DOI:** 10.3390/s19235331

**Published:** 2019-12-03

**Authors:** Prasertsak Tiawongsombat, Mun-Ho Jeong, Alongkorn Pirayawaraporn, Joong-Jae Lee, Joo-Seop Yun

**Affiliations:** 1 Electronics Engineering Technology, College of Industrial Technology, King Mongkut’s University of Technology North Bangkok, 1518 Pracharad 1 Rd., Wongsawang, Bangsue, Bangkok 10800, Thailand; prasertsak.t@cit.kmutnb.ac.th; 2 Division of Robotics, Kwangwoon University, 20 Gwangun-ro, Nowon-gu, Seoul 01897, Korea; 3 Department of Control and Instrumentation Engineering, Kwangwoon University, 20 Gwangun-ro, Nowon-gu, Seoul 01897, Korea; alongkorn.kmutnb@gmail.com; 4 Center of Human-centered Interaction for Coexistence, 5, Hwarang-ro 14-gil, Seongbuk-gu, CHIC, Seoul 02792, Korea; arbitlee@chic.re.kr; 5 Mechatronics Technology Convergence R&D Group, Korea Institute of Industrial Technology, 320 Techno Sunhwan-ro, Yuga-eup, Dalseong-gun, Daegu 42994, Korea; jsyun@kitech.re.kr

**Keywords:** human–robot interaction, attention model, measure of attentiveness, relevance theory, Scalable Hidden Markov Model

## Abstract

Attention capability is an essential component of human–robot interaction. Several robot attention models have been proposed which aim to enable a robot to identify the attentiveness of the humans with which it communicates and gives them its attention accordingly. However, previous proposed models are often susceptible to noisy observations and result in the robot’s frequent and undesired shifts in attention. Furthermore, most approaches have difficulty adapting to change in the number of participants. To address these limitations, a novel attentiveness determination algorithm is proposed for determining the most attentive person, as well as prioritizing people based on attentiveness. The proposed algorithm, which is based on relevance theory, is named the Scalable Hidden Markov Model (Scalable HMM). The Scalable HMM allows effective computation and contributes an adaptation approach for human attentiveness; unlike conventional HMMs, Scalable HMM has a scalable number of states and observations and online adaptability for state transition probabilities, in terms of changes in the current number of states, i.e., the number of participants in a robot’s view. The proposed approach was successfully tested on image sequences (7567 frames) of individuals exhibiting a variety of actions (speaking, walking, turning head, and entering or leaving a robot’s view). From these experimental results, Scalable HMM showed a detection rate of 76% in determining the most attentive person and over 75% in prioritizing people’s attention with variation in the number of participants. Compared to recent attention approaches, Scalable HMM’s performance in people attention prioritization presents an approximately 20% improvement.

## 1. Introduction

Attention is a process involving human factors. Human factors play a central role in the attentiveness determination process, especially when qualitative information and uncertainties are involved [1,2,3,4,5]. Intuitively, attention is an essential process for starting social interaction between human beings. To begin giving attention in a social interaction, a person with whom to communicate must first be identified. Most people perform this selection subconsciously, i.e., they identify who, from those in a given room or group, is worthy of their attention. Likewise, an intelligent service robot has to select a person in the group before their bi-directional communication starts. Therefore, the robot is required to possess attention-selecting capabilities as a fundamental function based on human social expectations; when the robot is equipped with such capabilities, people can interact with it in the same way that they interact with other people [6,7,8,9,10,11]. However, humans often stay in groups instinctively for communication. Before starting a conversation, the speaker evaluates to their prospective communicators and selects one from among them with whom to communicate. For this reason, when the service robot communicates in a multi-person interaction, it also evaluates and prioritizes the attention of the prospective communicators members individually, in terms of their perceived attentiveness; this process is called attention prioritization. The robot then selects the person who has the highest attentiveness (i.e., the most attentive person) to be the person with whom it communicates.

Most attention systems are generally composed of two distinctive sections: (1) feature extraction and (2) an attention model. (1) Feature extraction extracts attention-related visual features (ostensive-stimuli) from an image sequence and/or audio features from a sound stream. Various visual features are often chosen to be used as stimuli for the attention system such as the distance between a robot and a person [12,13], the head direction of the people participating in an interaction [14,15,16,17,18,19], and/or visual speaking status detection [20,21,22,23,24,25]. When audio features are used for the attention model, the direction of a sound source and the distance to a sound source are usually adopted [26,27,28]. (2) The attention model evaluates the selected stimuli and computes the attentiveness of each person. Finally, the most attentive person, as well as the attention priority of the individuals, in terms of their attentiveness, are determined. Intuitively, a robot equipped with an attention system can be considered to be more flexible and effective in their interactions with humans compared to the robot without one.

Overall, most previous methods have employed either a set of event conditions, heuristic equations, or both, such that the methods operate under predefined parameters and rules. However, the heuristic approaches presented in the literature are often susceptible to noisy observations and may produce frequent undesired attention shifts by the robot. Furthermore, their performance also suffers when they must contend with changes in the number of persons and observations, as they have difficulty adapting the state numbers accordingly in real-time.

To overcome such difficulties, a novel attentiveness determination approach based on relevance theory [29] is introduced. The relevance theory describes how humans communicate with each other and how a person evaluates the attention of other people during interaction exchanges. Thus, this theory was applied and converted to a mathematical form that aims to determine the most attentive person and prioritize people according to their relative attentiveness. Thus, a model was developed which aims to determine the most attentive person and prioritize people according to their relative attentiveness. The proposed approach consists of (1) a Scalable Hidden Markov Model (Scalable HMM) for attentiveness determination and (2) a probabilistic approach to compute the relevance for stimuli. The Scalable HMM has a scalable number of states and observations, and online adaptability for state transition probabilities with respect to changes in the current number of states. To test the proposed approach, the Scalable HMM was applied to 10 image sequences (7567 frames) of individuals exhibiting a variety of actions (speaking, walking, turning head, and entering or leaving a robot’s view). The detection rates achieved by the proposed approach, for both determination of the most attentive person and for people attention prioritization, were obtained and compared to those by recent robot attention model approaches.

The remainder of the paper is organized as follows. Section 2 reviews related research. Section 3 introduces a probabilistic stimuli-relevance computation approach based on relevance theory. The Scalable HMM-based attentiveness determination method is described in Section 4. Experimental results and the conclusions are discussed in Section 5 and Section 7, respectively.

## 2. Related Work

In the past decade, several researchers have integrated psychological studies into robotics research. Such works have estimated the mental states of other people by observing their behaviors and aimed to design a robot with human-like attention capabilities [30,31,32,33,34]. Psycholinguistic studies revealed that speaking status plays an important role in attention, in that a listener’s visual attention is driven by what they hear [35,36]. As a result, the speaking status is usually considered as a fundamental feature of a robot’s attention system [37,38,39,40,41,42]. Robot attention models can be categorized into two groups: those which rely on fixed rules and those which rely on arithmetic equations. When fixed rules are employed based on a logical set of event conditions, the satisfaction of a given measure leads to the model selecting a person as the most attentive person. Use of adopted arithmetic equations involves computation of the attentiveness of each person; finally, people prioritization can be determined by a comparison of the computed attentiveness.

In an approach that utilized a set of event conditions based on locations of a sound source and human face [37], an attention system was proposed for receptionists and companion robots. The system operated under the assumption that there is a single sound source at a time. The rules for the selection of the most attentive person are defined as follows: (1) if the location difference between a located sound source and a detected human face in the robot’s view is within $\pm 10^{\circ }$, the system associates the sound source with the human face. The person belonging to the associated face is determined as the most attentive person; (2) if the location difference changes such that it exceeds $\pm 30^{\circ }$ for three seconds, the system dissociates the sound source from that detected face, and the robot then loses its focus on the most attentive person; (3) step (1) and (2) are repeated.

A few years later, a focus of attention (FOA) system based on a detected speaking person was proposed [38]. Their method applied a multi-modal anchoring [39] for tracking a person of interest (POI). The only speaking person, who is facing the robot, can assume the role of POI. The event conditions are as follows: (1) a robot determines a speaking person as the POI (i.e., the most attentive person); (2) as long as the speech of the POI is anchored, other speaking people are ignored; (3) when the POI stops speaking for more than two seconds, the POI loses its speech anchor and another person can become the POI; (4) if no other person appearing in the robot’s view is speaking, the previous POI remains the most attentive person.

POI selection based on the gazing direction of a human face and a sound source location [40] was presented. However, people attention prioritization cannot be achieved in this case. In short, the face direction validates detected sounds as voices and the robot only gives attention to a person facing the robot. The logical set of event conditions for selecting the most attentive person is as follows: (1) there is a person facing a robot; (2) a sound source is located and associated to the detected face; (3) the person associated with the detected face and sound becomes the speaking person and the POI.

A parameter of intimacy to determine the selection priority of an interactive partner using interaction distance was also proposed [41]. This proposed method is based on the concept of proxemics for communication between a robot and multiple people. Proxemics suggests that the more intimate the communication, the nearer the target person stands. Interaction distance is roughly classified into four groups: intimate distance, personal distance, social distance, and public distance. A person with the highest intimacy is determined as the most attentive person for an interaction, with parameters of the intimacy equation pre-defined heuristically.

A value representing the attentiveness of a person was presented by Bennewitz et al. [42]. This value is computed by a weighted sum of three multimodal factors where the weights are constant and heuristically decided. Three factors are: (1) the time when the person last spoke, (2) the distance of the person to the robot (estimated according to the size of a bounding box around a person’s face), and (3) the person’s location relative to the front of the robot. The person with the highest value is determined as the most attentive person and is given the robot’s focused attention of attention of the robot. Attention prioritization is then simply achieved by sorting the magnitude of the computed values.

## 3. Introduction to Relevance Theory and Attention Model

This section introduces the concept of the relevance of observed features (i.e., stimuli-relevance) for attentiveness evaluation. Unlike previous attention approaches that employ heuristic parameters to calculate the attentiveness values of people, the stimuli-relevance values are computed probabilistically. Particularly, the proposed approach is derived based on a psychological theory of human communication methodology, called relevance theory. With this approach, a robot may evaluate attention as a person does during an interaction.

### 3.1. Relevance Theory in Multiple People-to-Robot Interactions

Relevance theory [29] explains a method of human communication that takes into account implicit inferences. Inferential communication not only intends to affect the thoughts of an audience but also seeks to elicit recognition from the audience that the communicator has an intention.

The method argues that individuals who engage in communication usually have the same notion of relevance in mind. To determine the most relevant communicator, an audience (in case of, a robot) searches for a certain meaning in any given communication situation and stops processing the situation when a meaning that fits the audience member’s expectation of relevance is found (i.e., the communicator with maximum relevance is identified).

**Figure 1:**
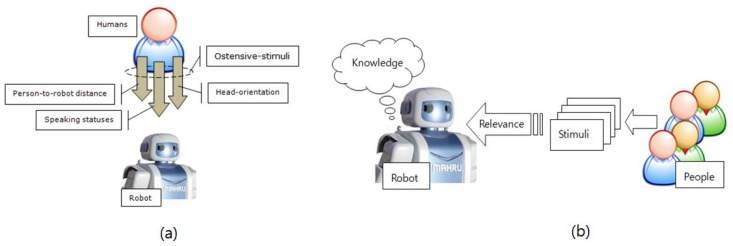
Ostensive-stimuli in people-to-robot interaction: (**a**) A robot and ostensive-stimuli of a single person; (**b**) relevance acquisition from observed ostensive-stimuli of multi-person with respect to robot’s equipped knowledge.

In human–human interaction, the ostensive-stimulus is an act by a human, produced during the interaction, which is appealing and mutually manifests between people. Ostensive-stimuli must satisfy two conditions: (1) they must attract the audience’s attention, and (2) they must allow the attention of the audience to be focused on the communicator’s intentions. In this work, the people-to-robot interaction (MPRI) can be illustrated as shown in Figure 1.

Figure 1a depicts the robot perceiving ostensive-stimuli from a single person (i.e., the person-to-robot distance, the head-orientation, and the speaking statuses). Figure 1b shows an overview of the robot acquiring the relevance of ostensive-stimuli based on its equipped knowledge to understand people intentions. Particularly, let us clarify this situation as follows:

- A robot is the only audience.
- *N* persons possess *N* different levels of intention that should be understood by the robot.
- The intention of any person is to start communication with the robot and become the most attentive person.
- Human’s ostensive stimuli are assumed to be confined to person-to-robot distance, head-orientation, and speaking statuses.
- The robot simultaneously receives *N* intentions of people, evaluated from all relevant ostensive stimuli, and searches for the person with the maximum relevance in terms of intention.

**Figure 2:**
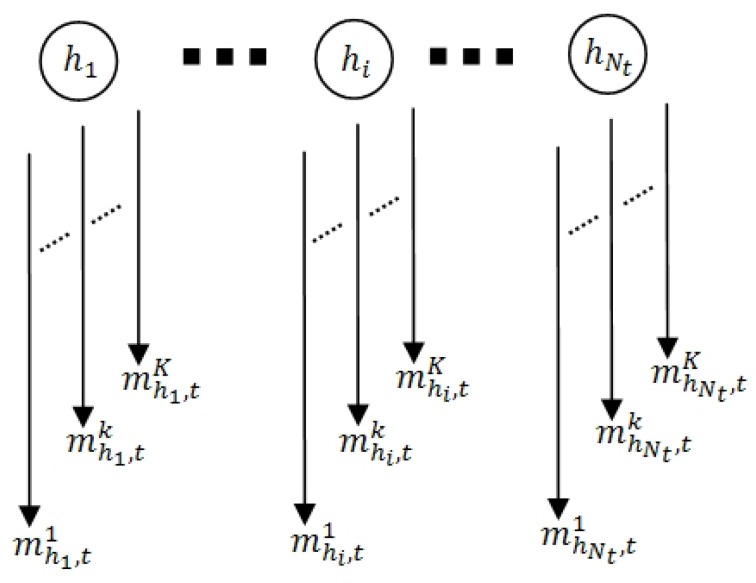
Participating people $\left\{h_{i}\right\}$ as sources of the ostensive-stimuli $m_{h_{i},t}^{k}$, where $1\leq i\leq N_{t}$ and 1≤k≤K. $N_{t}$ implies the number of participants at time *t*, and *K* denotes the number of ostensive-stimuli.

Hence, Figure 1 can be represented as the diagram of MPRI, as shown in Figure 2, in which the people can be considered as the sources of ostensive-stimuli.

Let us define a group of participants as $\left\{h_{i}\right\}=\left\{h_{1},h_{2},\ldots ,h_{N_{t}}\right\}$, where $N_{t}$ is the number of participants at time *t* and $1\leq i\leq N_{t}$. Focusing on any person $h_{i}$, $m_{h_{i},t}^{k}$ are the observed ostensive-stimuli, which are scalar, independent, and bounded with their observation ranges. $m_{h_{i},t}={\left[m_{h_{i},t}^{1},\ldots ,m_{h_{i},t}^{k},\ldots ,m_{h_{i},t}^{K}\right]}^{T}$ denotes an ostensive-stimuli vector of person $h_{i}$ at *t*, where *K* is the number of ostensive-stimuli and 1≤k≤K. Hence, $o_{t}={\left[m_{h_{1},t}^{T}\ldots m_{h_{i},t}^{T}\ldots m_{h_{N_{t}},t}^{T}\right]}^{T}$ becomes an ostensive-stimuli vector of a group of participants at *t*. Later, Section 4.1.1 describes probabilistic stimuli-relevance based on the relevance theory.

### 3.2. Attention Model’s Structure

Attention model algorithms presented in past literature mostly involve algorithms with heuristic parameters for the determination of the most attentive person. As the use of fixed parameters can result in frequent undesired changes of states (i.e, undesired attention shifts) in situations with noisy observations. A probabilistic approach may be better suited to robot attention models.

Previously presented heuristic attention approaches simply define a detected speaking person as the most attentive person. Although it seems natural to employ the speaking status as the most influential stimulus, defining the speaker as the most attentive person over-emphasizes the importance of speaking, i.e., the selection of a person can be impacted by false detection of speaking status and should involve other stimuli, such as the person’s head pan or the person’s distance from the robot. The proposed approach differs from such heuristic approaches in that it considers multiple stimuli to order attentiveness, rather than solely speaking status.

The proposed probabilistic method focuses on improving the performance of determining the most attentive person and prioritizing people based on their attentiveness. To do so, both effective computation of attentiveness and adaptation to the changes in the number of participants (i.e., communicators) and observations (i.e., changes in person-to-robot distances, head pans, and speaking statuses) are taken into account. Three ostensive-stimuli are considered: person-to-robot distance, head pan of a person, and speaking status. Particularly, person-to-robot distance and the head pan of a person are treated as typical observations for computation of stimuli-relevance probabilities (Section 4.1.1). Speaking status is used for determining adaptable state transition probabilities in run-time (Section 4.1.2). Hence, with adjustable state transition probabilities, more flexible and efficient attentiveness determination, for a robot attention model, can be achieved.

The model’s capability of coping with a change in the number of observations is useful for some situations, such as those with several associated observations, which may be occasionally inaccessible or unimportant during operation. In such situations, by temporarily and effectively scaling down the number of observations, the proposed approach can still robustly compute the probabilities of observations. In this way, computational failure during run-time can be avoided. Furthermore, when the previously missing observations become available again, the approach simultaneously adapts its computation process of probabilities of observations according to the current number of observations and states. This issue is discussed in detail in Section 4.2.

## 4. Attentiveness Determination Using Scalable Hidden Markov Model

**Figure 3:**
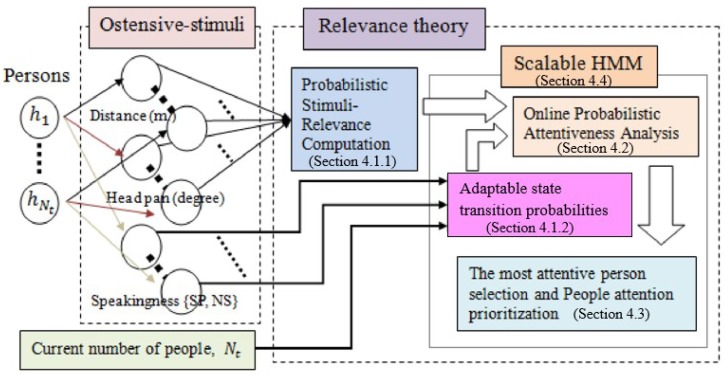
Attention model for the most attentive person selection and people attention prioritization based on relevance theory. HMM = Hidden Markov Model; NS = non-speaking status; SP = speaking status.

In this section, a Scalable HMM, based on the relevance theory, for attentiveness determination is described. The Scalable HMM recalls a similar approach [43]. Both the proposed model and the dynamic HMM are able to handle changes in the number of states during run-time. Differently, our proposed Scalable HMM is also capable of coping with changes in the number of observations attributed to changes in the number of states. Figure 3 depicts the main processes of the proposed approach in five parts. First, the probabilistic attentiveness computation based on stimuli-relevance (Section 4.1) presents the method by which the stimuli-relevance probabilities are computed using the three ostensive-stimuli (distance from person-to-robot, angle of a person’s head pan and a person’s speaking status). Next, an online probabilistic attentiveness analysis (Section 4.2) demonstrates the probabilistic computation between the previous and current states, for the case in which the number of detected persons changes. Section 4.3 explains how the most attentive person is selected and the attention prioritized in run-time. Finally, Section 4.4 describes how the Scalable HMM-based attention model is applied, using Particle Swarm Optimization (PSO), to the robot attention model.

### 4.1. Probabilistic Acomputation Based on Stimuli-Relevance

Probabilistic attentiveness computation from three ostensive-stimuli (introduced in Section 3.2) is thoroughly explained in two parts: (1) Section 4.1.1 describes how stimuli-relevance probabilities from two stimuli (a person-to-robot distance and angle of a head pan) are obtained; (2) Section 4.1.2 depicts the adaptable state transition probabilities, which are used to flexibly consider state transition probabilities in run-time and thus increase efficient attentiveness determination for the robot attention model. In consideration of state transition probabilities, a person’s speaking status, as well as the number of persons in camera view, are used to determine probabilistic attentiveness in run-time. Then, the probabilistic attentiveness is used to determine the most attentive person and arrange people attention prioritization, as discussed in the following sections.

#### 4.1.1. Probabilistic Stimuli-Relevance Computation

As discussed in Section 3.1, an ostensive-stimulus both attracts the attention of a robot and tries to convey to the robot the meaning intended by the communicators. Because ostensive-stimuli are noisy, the probabilistic approach is considered as a potentially superior alternative to computing the stimuli-relevance of a given person.

In particular, human’s relevance computation here consists of two fundamental properties. The first is the attraction of ostensive-stimuli, wherein a person’s intention to communicate is conveyed through the emission of stimuli in the form of probability density function (pdf). The other is restraint of ostensive-stimuli, wherein a person has no intention to emit particular stimuli, and is also in the form of pdf. Considering $m_{h_{i},t}$ and the ostensive-stimuli vector of any person $h_{i}$, the probabilities of attraction, $P_{i}\left(m_{h_{i},t}\right)$, and restraint, ${\bar{P}}_{i}\left(m_{h_{i},t}\right)$, can be defined by

(1) $$\begin{array}{ccc} P_{i}\left(m_{h_{i},t}\right) & = & \prod_{k=1}^{K}c_{k}\left(m_{h_{i},t}^{k}\right), \end{array}$$

(2) $$\begin{array}{ccc} {\bar{P}}_{i}\left(m_{h_{i},t}\right) & = & \prod_{k=1}^{K}{\bar{c}}_{k}\left(m_{h_{i},t}^{k}\right), \end{array}$$

where $c_{k}\left(m_{h_{i},t}^{k}\right)$ and ${\bar{c}}_{k}\left(m_{h_{i},t}^{k}\right)$ denote the attraction and the restraint distributions of the *k*th ostensive-stimulus, respectively.

From cognitive psychology, attention is the behavioral and cognitive process of selectively concentrating on a discrete aspect of information [44], whether deemed subjective or objective, while ignoring other perceivable information. Building on this, we define a state variable, $q_{t}\in \left\{s_{1},\ldots ,s_{i},\ldots ,s_{N_{t}}\right\}$, where a person $h_{i}$ has an intention to start communication with the robot while others have no intention to do it. Consider $\left\{h_{i}\right\}$ as a group of participating people. Using Equations (1) and (2), the probability of relevance of the ostensive-stimuli of given a state $s_{i}$, $P(o_{t}|q_{t}=s_{i})$, is defined as follows:

(3) $$P\left(o_{t}|q_{t}=s_{i}\right)=P_{i}\left(m_{h_{i},t}\right)\prod_{j\neq i}{\bar{P}}_{j}\left(m_{h_{j},t}\right),\;\;\;\;\;1\leq i,j\leq N_{t},\;i\neq j.$$

For example, if the current number of participating people is $N_{t}$, Equation (3) becomes:

(4) $$\begin{array}{c} P\left(o_{t}|q_{t}=s_{1}\right)=P_{1}\left(m_{h_{1},t}\right){\bar{P}}_{2}\left(m_{h_{2},t}\right)\ldots {\bar{P}}_{N_{t}-1}\left(m_{h_{N_{t}-1},t}\right){\bar{P}}_{N_{t}}\left(m_{h_{N_{t}},t}\right)\\ P\left(o_{t}|q_{t}=s_{2}\right)={\bar{P}}_{1}\left(m_{h_{1},t}\right)P_{2}\left(m_{h_{2},t}\right)\ldots {\bar{P}}_{N_{t}-1}\left(m_{h_{N_{t}-1},t}\right){\bar{P}}_{N_{t}}\left(m_{h_{N_{t}},t}\right)\\ \;\;\;\;\;\;\;\;\;\;\;\;\;\;\;\;\;\;\;\;\;\;\;\;\;\;\;\;\vdots \\ P\left(o_{t}|q_{t}=s_{N_{t}}\right)={\bar{P}}_{1}\left(m_{h_{1},t}\right){\bar{P}}_{2}\left(m_{h_{2},t}\right)\ldots {\bar{P}}_{N_{t}-1}\left(m_{h_{N_{t}-1},t}\right)P_{N_{t}}\left(m_{h_{N_{t}},t}\right). \end{array}$$

Note that Equations (1)–(3) illustrate good scalability in the sense that $P(o_{t}|q_{t}=s_{i})$ adapts efficiently with respect to the number of participating people and observations in run-time. As a result, effective computation of attentiveness of people can be achieved.

#### 4.1.2. Online Adaptable State Transition Probabilities

This section introduces online adjustable state transition probabilities based on the speaking statuses of participants. Speaking statuses of persons are used as the conditional parameter in the model. Furthermore, the current number of participants are also taken into account. Hence, an effective and improved computation of attentiveness can be achieved in terms of sensitivity and adaptability with respect to speakers and changes in the number of participants.

Let us denote $Y_{h_{j},t-1}\in \left\{\mathrm{NS},\mathrm{SP}\right\}$ as the speaking statuses of person $h_{j}$ at time t−1, where NS is the non-speaking status and SP is the speaking status. The state transition probability distribution given a person’s speaking status, $P(q_{t}=s_{j}|q_{t-1}=s_{i},Y_{h_{j},t-1})$, is denoted by

(5) $$P\left(q_{t}=s_{j}|q_{t-1}=s_{i},Y_{h_{j},t-1}\right)=\left\{\begin{array}{cc} \frac{\rho_{\mathrm{ns}}}{R} & \mathrm{if}\;i=j,Y_{h_{j},t-1}=\mathrm{NS};\\ \frac{\rho_{\mathrm{sp}}}{R} & \mathrm{if}\;i=j,Y_{h_{j},t-1}=\mathrm{SP};\\ \;\frac{1}{R} & \mathrm{if}\;i\neq j,Y_{h_{j},t-1}=\mathrm{NS}\;\mathrm{or}\;\mathrm{SP}, \end{array}\right.$$

where $\rho_{\mathrm{ns}}$ and $\rho_{\mathrm{sp}}$ define the sensibility parameters of the attention model, influencing the sensitivity of the robot’s attention shifts with regard to the speaking and non-speaking persons during run-time. Further, $N_{t-1}\times N_{t-1}$ is the dimension of the state transition matrix.

The state transition matrix is now designed such that the transition to the same person (state) is $\rho_{\mathrm{ns}}$ or $\rho_{\mathrm{sp}}$ times more likely than the transitions to other persons, conditioned according to the previous speaking status of that person, whether it was non-speaking or speaking, respectively. Note also that $1\leq \rho_{\mathrm{ns}}<\rho_{\mathrm{sp}}$, and R is a normalizing constant used to ensure that each row of the state transition matrix sums to 1.

### 4.2. Online Probabilistic Attentiveness Analysis

Relevance theory states that a person retrieves relevance assumptions stored in their memory (knowledge of ostensive-stimuli with respect to a situation) and processes them with an inferential procedure to draw a conclusion.

To implement this procedure into the robot’s attention model in a similar manner, the inferential procedure can be mathematically emulated by statistical inference. The inference is performed by using quantitative data. A greater informativeness quantity results in better accuracy of inference.

For the analysis of attentiveness of a person $h_{j}$ until the current time *t*, we consider the probability of relevance of the partial observation sequence until time *t*, $O_{1:t}=\left\{o_{1}\;o_{2}\ldots o_{t}\right\}$ and state $q_{t}=s_{j}$ given a robot’s attention model λ. This yields:

(6) $$\alpha_{t}\left(j\right)=P\left(o_{1}\;o_{2}\ldots o_{t},q_{t}=s_{j}|\lambda \right).$$

Efficiently, we can solve for $\alpha_{t}\left(j\right)$ from Equation (6) inductively, as follows:

(1) Initialization
  (7) $$\begin{array}{c} \alpha_{1}\left(i\right)=\pi_{i}\;P\left(o_{1}|q_{1}=s_{i}\right),\;\;\;\pi_{i}=\frac{1}{N_{1}},\;1\leq i\leq N_{1}. \end{array}$$
(2) Induction
  (2.1) Validation
    - Checking the current number of participants.
    - Iterating over states at t−1 and *t* for states comparison and validation.
    Correcting state indexes, if required.
  (2.2) Computation
    case 1: $N_{t-1}=N_{t}$
    (8) $$\begin{array}{c} \alpha_{t}\left(j\right)=\sum_{i=1}^{N_{t}}\left[\alpha_{t-1}\left(i\right)a_{ij}\right]\;P\left(o_{t}|q_{t}=s_{j}\right),\;\;\;1\leq j\leq N_{t}, \end{array}$$
    case 2: $N_{t}>N_{t-1}$
    (9) $$\begin{array}{c} \alpha_{t}\left(j\right)=\left\{\begin{array}{c} \sum_{i=1}^{N_{t-1}}\left[\alpha_{t-1}\left(i\right)a_{ij}\right]\;P\left(o_{t}|q_{t}=s_{j}\right),\;\;\;1\leq j\leq N_{t-1},\\ \pi_{j}\;P\left(o_{t}|q_{t}=s_{j}\right),\;\;\;\pi_{j}=\frac{1}{N_{t}},\;N_{t-1}<j\leq N_{t} \end{array}\right. \end{array}$$
    case 3: $N_{t}<N_{t-1}$
    (10) $$\begin{array}{c} \alpha_{t}\left(j\right)=\sum_{i=1}^{N_{t-1}}\left[\alpha_{t-1}\left(i\right)a_{ij}\right]\;P\left(o_{t}|q_{t}=s_{j}\right),\;\;\;1\leq j\leq N_{t}, \end{array}$$
    where $\pi =\{\pi_{i}\}$ is the initial state distribution of Scalable HMM for the attention model, and $a_{ij}=P\left(q_{t}=s_{j}|q_{t-1}=s_{i},Y_{h_{j},t-1}\right)$ are the state transition probabilities.

**Figure 4:**
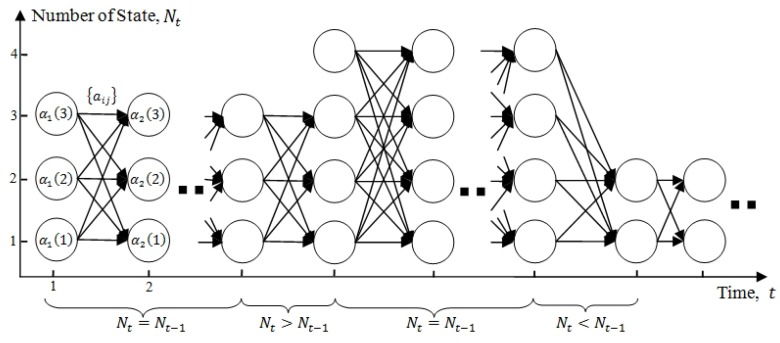
Illustration of the implementation for $\alpha_{t}\left(i\right)$ computation in terms of a lattice of observations and states during different cases: $N_{t}=N_{t-1}$, $N_{t}>N_{t-1}$, and $N_{t}<N_{t-1}$.

Figure 4 illustrates the induction procedure, showing how state $s_{j}$ can be reached at time *t* from the $N_{t-1}$ possible states at previous time t−1. Prior to $\alpha_{t}\left(j\right)$ computation, the validation process (Step (2.1)) must be performed at each induction step. The process, which has $O(N^{2}K)$ computational complexity, validates the current number of states (the participants in a robot’s view). In the case of a decrease in the number of participants, an index re-correction of the participants may be required, such that computation failure can be avoided in run-time.

### 4.3. Online Most Attentive Person Selection and People Attention Prioritization

For selecting the most attentive person and prioritizing people based on their attentiveness, the proposed attention model first evaluates the probabilistic stimuli-relevance of participants. Next, the probabilistic attentiveness of each person, $\alpha_{t}\left(j\right)$, is computed.

Finally, the most attentive person, denoted by $q_{t}^{*}$, is determined as the person with the maximum attentiveness. Hence, $q_{t}^{*}$ is denoted by

(11) $$\begin{array}{ccc} q_{t}^{*} & = & \underset{1\leq j\leq N_{t}}{\mathrm{argmax}}\left[P\left(q_{t}=s_{j}|O_{1:t}\right)\right]\\ & = & \underset{1\leq j\leq N_{t}}{\mathrm{argmax}}\left[P\left(O_{1:t},\;q_{t}=s_{j}\right)/P\left(O_{1:t}\right)\right]\\ & = & \underset{1\leq j\leq N_{t}}{\mathrm{argmax}}\left[P\left(O_{1:t},\;q_{t}=s_{j}\right)\right]\\ & = & \underset{1\leq j\leq N_{t}}{\mathrm{argmax}}\left[\alpha_{t}\left(j\right)\right]. \end{array}$$

By comparing $\left\{\alpha_{t}\left(j\right)\right\}$ for current participants at *t*, the prioritization of participants with respect to their attentiveness can be achieved.

### 4.4. Learning Approach for Scalable HMM-Based Attention Model Using Particle Swarm Optimization

In general, the HMM parameters are estimated using the Baum–Welch algorithm [45]. However, it is well known that the Baum–Welch algorithm easily converges to local optimum solutions. To find the global solution or better optimum solutions, estimating HMM parameters using Particle Swarm Optimization (PSO) [25,46,47] has been an alternative method, showing superior results compared to the conventional Baum–Welch method. Further, the PSO algorithm also provides a simple method for solving complex optimization problems. Therefore, we apply a training approach based on PSO for our robot attention model.

The PSO-based learning approach is briefly introduced in this section. Let us denote $\psi =\;\{\rho_{\mathrm{ns}},\rho_{\mathrm{sp}},\left\{\mu_{k},{\bar{\mu }}_{k}\right\},\left\{\sigma_{k}^{2},{\bar{\sigma }}_{k}^{2}\right\}\}$ as a vector of system parameters to be estimated, where $\left\{\mu_{k},{\bar{\mu }}_{k}\right\}$ and $\left\{\sigma_{k}^{2},{\bar{\sigma }}_{k}^{2}\right\}$ are the means and variances of attraction and restraint distributions of ostensive-stimuli, 1≤k≤K, respectively.

In the PSO-based learning approach, the model is encoded into a string of real numbers. The vector ψ acts as a particle, representing the position vector $x_{i}$. With each position vector $x_{i}$, there is an associate velocity vector $v_{i}$, modeling the capacity of the particle to move from a given position $x_{i}^{z}$ at the *z*th iteration to another position $x_{i}^{z+1}$ in a successive iteration of the space solution sampling process.

The initial positions $X^{0}=\left\{x_{i}^{0};i=1,2,\ldots ,N_{p}\right\}$ and their velocities $V^{0}=\left\{v_{i}^{0};i=1,2,\ldots ,N_{p}\right\}$ of $N_{p}$ particles of the swarm can be randomly generated [48]. The ranges can differ for different dimensions of particles.

The degree of optimality of each particle is evaluated at the *z*th iteration by computing its logP(O|ψ). The fitness function is defined as follows:

(12) $$\begin{array}{c} \;\;\;\;\;\;\;\;\;\;\;\;P\left(O^{m}|\psi \right)=\sum_{j=1}^{N}\alpha_{T}^{m}\left(j\right) \end{array}$$

(13) $$\begin{array}{c} f\left(x_{i}\right)=\log P\left(O|\psi \right)=\frac{1}{M}\sum_{m=1}^{M}\log P(O^{m}|\psi ), \end{array}$$

where $O^{m}=\left\{o_{1}^{m}\;o_{2}^{m}\ldots o_{T}^{m}\right\}$ is the $m^{\mathrm{th}}$ observation sequence. The previous best particle, **pbest**, storing the best position that has been reached up to now by the *i*th particle, is found by ${\mathrm{pbest}}_{i}^{z}={\mathrm{argmax}}_{1\leq h\leq z}\left[f(x_{i}^{h})\right]$. Next, the global best particle, **gbest**, which is the optimum position in the overall swarm, can be computed by ${\mathrm{gbest}}^{z}={\mathrm{argmax}}_{1\leq i\leq N_{p}}\left[f(x_{i}^{z})\right]$.

The velocity of the $d^{\mathrm{th}}$ of each particle is updated with dynamic inertia as follows:

(14) $$\begin{array}{c} v_{i,d}^{z+1}=w^{z}v_{i,d}^{z}+\phi_{1}r_{1}\left({\mathrm{pbest}}_{i,d}^{z}-x_{i,d}^{z}\right)+\phi_{2}r_{2}\left({\mathrm{gbest}}_{d}^{z}-x_{i,d}^{z}\right) \end{array}$$

(15) $$\begin{array}{c} w^{z}=w_{\max}-\frac{w_{\max}-w_{\min}}{{\mathrm{iter}}_{\max}}z, \end{array}$$

where $r_{1}$ and $r_{2}$ are two uniformly distributed random positive numbers, used to provide the stochastic weighting. *w* is the inertia weight, affecting the influence of the old velocity on the new velocity. $\phi_{1}$ and $\phi_{2}$ are constants, called cognition and social acceleration, respectively. Next, the particle position is then updated as follows:

(16) $$x_{i,d}^{z+1}=x_{i,d}^{z}+v_{i,d}^{z+1}.$$

The velocity and position updating, and the optimization process are stopped when the condition of termination is satisfied. Finally, **gbest** is assumed to be the optimum solution for the model.

The terminating condition is that the maximum number of iterations, ${\mathrm{iter}}_{\max}$, is reached ($z={\mathrm{iter}}_{\max}$) or the increase of the optimum fitness is below a given threshold (i.e., $|f\left({\mathrm{gbest}}^{z}\right)|-|f\left({\mathrm{gbest}}^{z-1}\right)|<threshold$).

## 5. Experiments

To evaluate the performance, the proposed method was tested with 10 image sequences consisting of a total of 7567 frames, displaying individuals who were speaking, walking, turning their head, and entering or leaving a robot’s view. None of the 10 image sequences were used during the training phase. The performance results were compared to the performance of a human and two widely used attention approaches: a set of event conditions [38] and a heuristic algorithm [42].

### 5.1. Experimental Setup

This section gives an overview of the experiment setup and experimental scenarios used to verify the performance of the proposed robot attention model. Multiple persons stood in front of the robot and far away from the robot, by 2–3 m. In our experiment, three persons were a suitable number for our devices to be able to observe them fully in the camera’s view while they were still in the range of sound communication. For all image sequences, three attention-related features (speaking status, the distance between a person and a robot, and a person’s head pan angle), were determined visually and automatically using approaches described in Appendix A. The proposed attentiveness computation model and all feature detection algorithms have been implemented using a PC equipped with a 3GHz Pentium 4 CPU, with 1GB of RAM, and an NVIDIA GeForce 6600.

**Figure 5:**
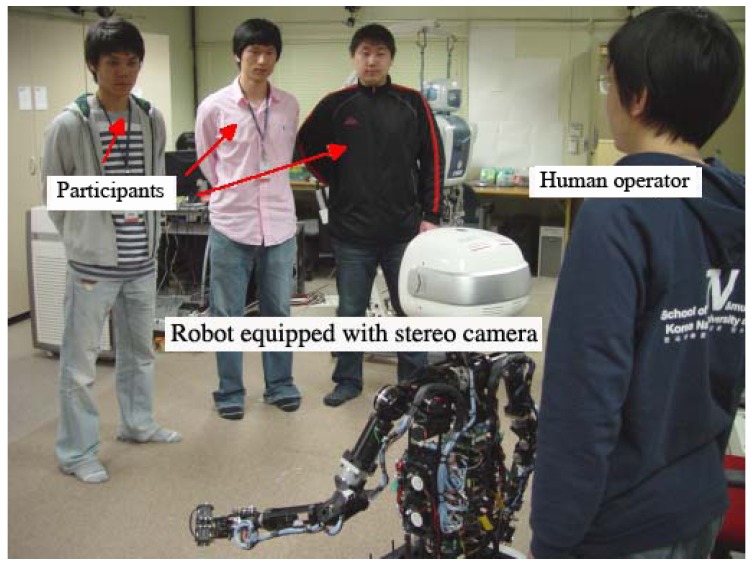
Our experimental setup.

Figure 5 illustrates the experiment setup; participating people stand in front of MAHRU-M, which has a Bumblebee2 stereo camera (www.ptgrey.com) attached on its head. MAHRU-M is a mobile humanoid robot platform based on a dual-network control system and coordinated task execution [49].

**Figure 6:**
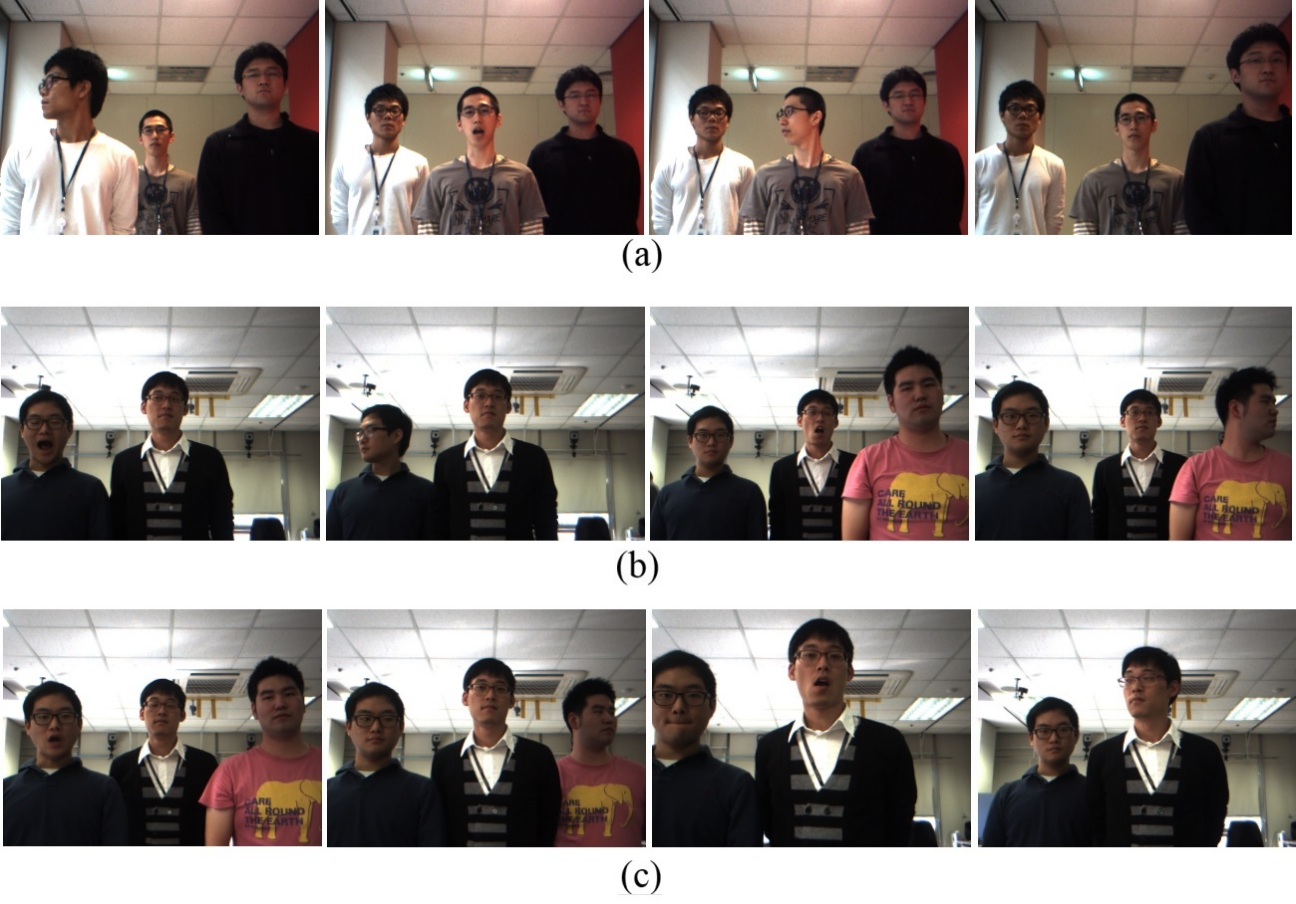
Sample images displaying individuals who exhibited speaking, walking, turning head, and entering/leaving a robot’s view.

Figure 6 illustrates various situations with participants randomly performing actions (speaking, head turning, walking toward or away from a robot, and entering or leaving a robot’s view). The first row (Figure 6a) shows an image sequence of three participants randomly turning their heads, exhibiting speaking or non-speaking intervals, and walking towards or away from the robot. The second and the third rows (Figure 6b,c, respectively) illustrate the image sequences, showing an individual entering or leaving the robot’s view.

**Figure 7:**
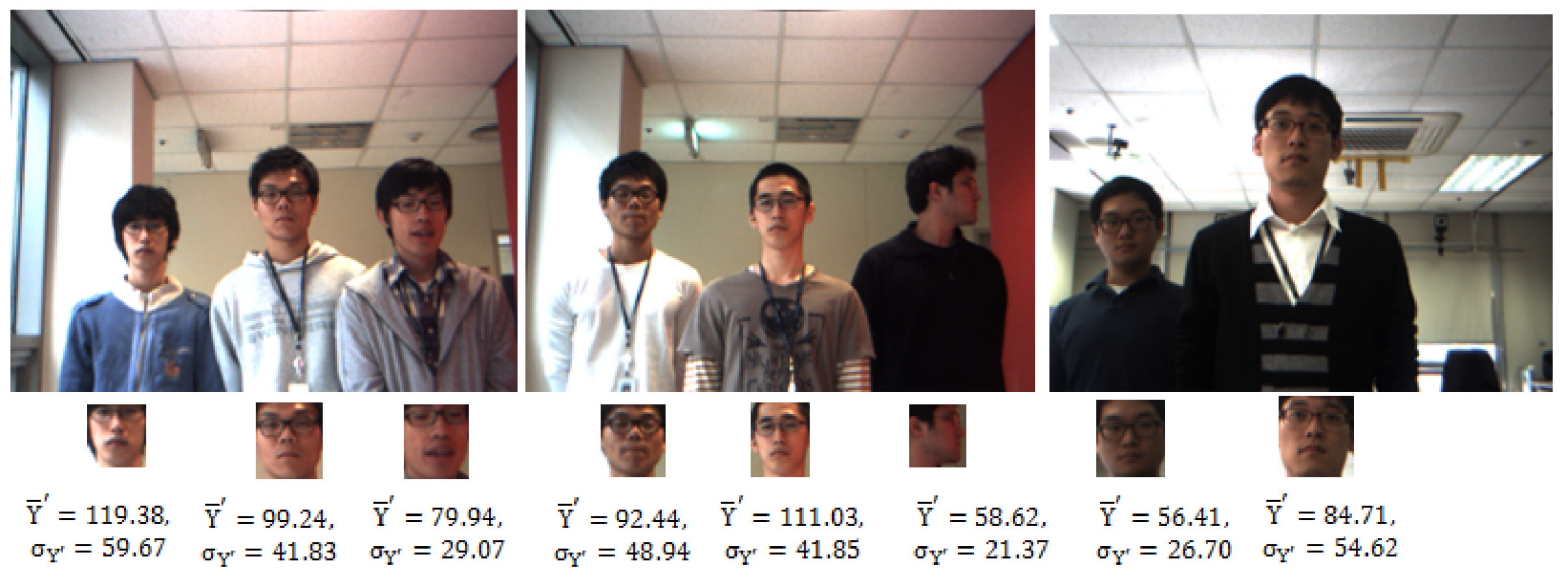
Face images of participants in different locations, sizes, and lighting conditions, in which (${\bar{Y}}^{\prime }$) is the mean of luma and its standard deviation ($\sigma_{Y^{\prime }}$).

The illumination conditions were natural and not controlled in any of the collected image sequences. As a result, the face size and the lighting conditions for the persons in different locations in the robot’s view differed, as shown in Figure 7. The illumination can be calculated and represented by the luma component (Y′) in Y′UV color space which is the weighted sum of RGB components of a color image. In Figure 7, the brightness and its variation of a face image of each person is demonstrated by the mean of luma (${\bar{Y}}^{\prime }$) and its standard deviation ($\sigma_{Y^{\prime }}$).

### 5.2. Attraction and Restraint Distributions of Ostensive-Stimuli

According to Figure 3, two ostensive-stimuli are involved in the computation of probabilistic stimuli-relevance in our attention model. Hence, the corresponding number of ostensive-stimuli is two (i.e., K=2; k=1 corresponds to the person-to-robot distance, and k=2 corresponds to head orientation (or head pan)). To design attraction and restraint distributions for each ostensive-stimulus, the stimuli must be analyzed with regard to their contributions to attentiveness.

Hence, we examined how much attention a person might give to someone who stands at different distances and looks in different directions. Intuitively, we assume that a person is likely to pay less attention to people who are further away or looking away, compared to those who are closer distances or looking directly at the person.

The attraction and restraint distributions for both the person-to-robot distance and the head pan angle can be reasonably designed by a folded normal distribution [50]. Conveniently, the folded normal distribution can be tuned such that it satisfactorily delivers the interpreted distribution’s characteristics of both stimuli, using the mean μ and variance $\sigma^{2}$ as parameters.

**Figure 8:**
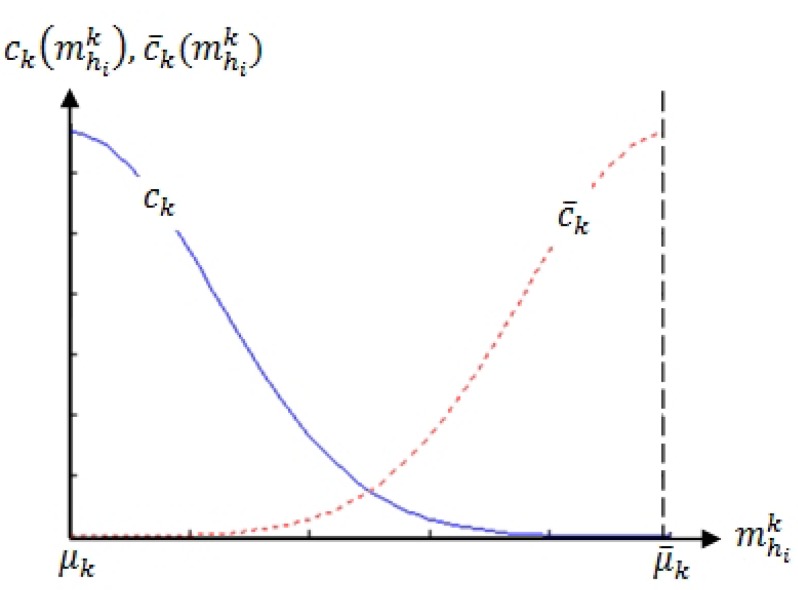
Probability distributions of attraction, $c_{k}$, and restraint, ${\bar{c}}_{k}$, of $k^{\mathrm{th}}$ ostensive-stimulus of person $h_{i}$ (k=1 indicates distance of person-to-robot and k=2 indicates head pan).

Figure 8 illustrates $\left\{c_{k},{\bar{c}}_{k}\right\}$, designed by the folded normal distributions, of the *k*th ostensive-stimulus of person $h_{i}$, in which $\left[\mu_{k},{\bar{\mu }}_{k}\right]$ denote the measuring scope of the *k*th ostensive-stimulus. Here, the measuring scopes of the person-to-robot distance and head-pan angles in our attention model were set to $\left[\mu_{1}=0.5\;m,{\bar{\mu }}_{1}=2.0\;m\right]$, and $\left[\mu_{2}=0^{\circ },{\bar{\mu }}_{2}=90^{\circ }\right]$, respectively.

## 6. Results

The human mind is a complex entity that represents a particular characteristic of people [51]. Every person has an individual opinion regarding who is the most attentive person during an interaction. Because of this, the common ground truth for determining the selection criteria for the most attentive person is difficult to practically determine.

Hence, to evaluate the proposed attention model, attention evaluation experiments were conducted with people. In these human evaluations of the most attentive person including attention prioritization were obtained. Ten users participated with the experiments on the same 10 image sequences used for testing the proposed attention model with the robot as the attention evaluator. They were asked to watch videos of image sequences of interacting people (see Figure 6), to evaluate who is the most attentive person, and prioritize people based on attentiveness. The users were also asked to consider only speaking statuses, distance, and head pan for the evaluation of attentiveness. Finally, for each image sequence, the most likely outcomes of the human evaluation were obtained by finding the maximum among user decisions.

The probabilities of detection ($P_{d}$), false alarm ($P_{f}$), and attention shift during intervals ($P_{s}$) were used as performance indicators. $P_{d}$ indicates the attention model’s performance regarding the detection of the most attentive person and the detection of people prioritization with respect to attentiveness. Let us denote $P_{d,m}$ as the ratio of the frames where the most attentive person is correctly detected to the total number of frames with the most attentive person. $P_{d,p}$ is the ratio of the frames where people’s attention is correctly prioritized to the total number of frames.

$P_{f,m}$ is the ratio of the frames where a person who is not the most attentive person is incorrectly detected as the most attentive person to the total number of frames where the given person is not the most attentive person. Finally, $P_{s}$ is calculated as follows:

(17) $$\begin{array}{c} P_{s,m}^{i}=\frac{\mathrm{number}\;\mathrm{of}\;\mathrm{transitions}\;\mathrm{from}\;\mathrm{MP}\;\mathrm{state}\;\mathrm{to}\;\neg \mathrm{MP}\;\mathrm{state}\;\mathrm{in}\;\mathrm{MP}\;\mathrm{intervals}}{\mathrm{number}\;\mathrm{of}\;\mathrm{MP}\;\mathrm{frames}}\\ P_{s,\neg m}^{i}=\frac{\mathrm{number}\;\mathrm{of}\;\mathrm{transitions}\;\mathrm{from}\;\neg \mathrm{MP}\;\mathrm{state}\;\mathrm{to}\;\mathrm{MP}\;\mathrm{state}\;\mathrm{in}\;\neg \mathrm{MP}\;\mathrm{intervals}}{\mathrm{number}\;\mathrm{of}\;\neg \mathrm{MP}\;\mathrm{frames}}\\ P_{s}=\sum_{i=1}^{N}\left[\frac{P_{s,m}^{i}+P_{s,\neg m}^{i}}{2}\right], \end{array}$$

where *N* is the total number of participants. MP and ¬MP refer to “the most attentive person” and “not the most attentive person,” respectively.

In the experiments with our proposed attention model, the model parameters were estimated with the PSO approach described in Section 4.4 using the training data (sequences of the observed ostensive-stimuli of people: speaking statuses, person-to-robot distance, and head-pan angle).

In the experiments with the two other attention methods, i.e., the set of event conditions [38] and the heuristic equations [42], the respective parameters of each approach were determined according to recommendations described in their studies. The same observations used in our proposed method were also used in these two approaches. A brief description of each approach is provided in Section 1.

**Figure 9:**
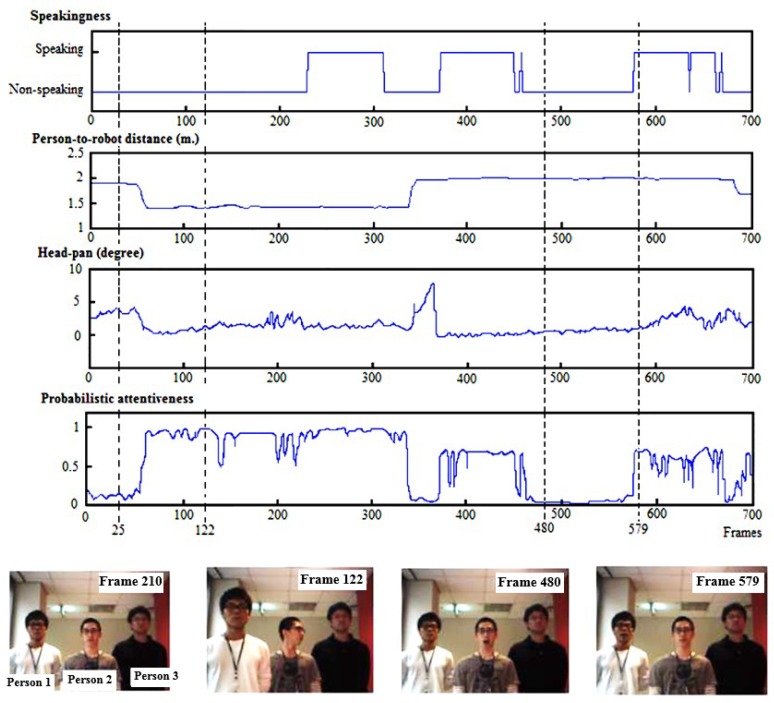
Observed attention-related features (ostensive-stimuli) of a single person (person 1) from one of the image sequences and his probabilistic attentiveness result.

Figure 9 demonstrates the observed ostensive-stimuli of a single person (person 1) from one of the image sequences. The first three graphs from the top depict the detected speaking status of person 1, the person’s estimated distance from the robot in meters, and the estimated head-pan angle in degrees over time, respectively. The fourth graph illustrates the probabilistic attentiveness result of person 1, computed by the proposed attentiveness computation model. Sample images on the right side of the figure illustrate the action sequences of people in this image sequence, in which the people are labeled as person no. 1, person no. 2, and person no. 3.

In frame 25, person no. 1 was approximately 1.9m away from the robot and was looking relatively straight at the robot. He had a respectively low attentiveness of 0.1185. Next, in frame 122, his attentiveness became higher (≈0.98) because he came closer to the robot (≈1.4m away). At frame 480, person no. 1 had a very low attentiveness of 0.012 because he was very far away from the robot (≈2m), even though he looked straight at the robot. However, his attentiveness rose gradually as he spoke, and his attentiveness became 0.7 at frame 579.

**Figure 10:**
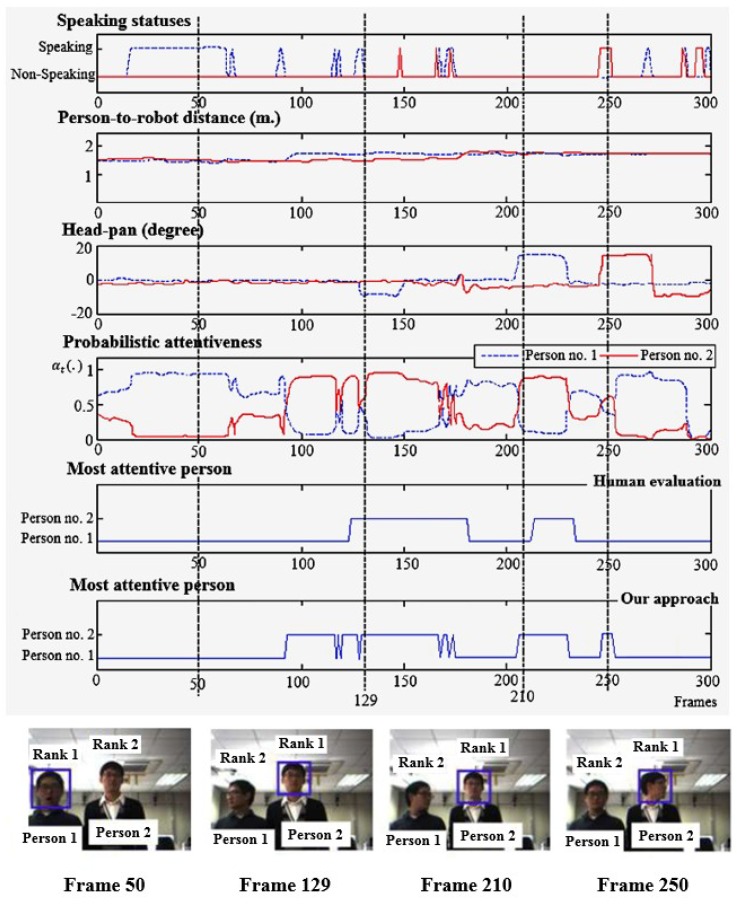
Robot’s attention results, from one of the image sequences, showing observed attention-related visual features, the most attentive person selection, and people attention prioritization outcomes. The most attentive person is indicated by a rectangle and the attentiveness ranking is indicated by the number on the top of each person.

Figure 10 illustrates the attention outcomes of one image sequence of a situation of two participants and the observed ostensive-stimuli of each person. The sample images of the sequence are also shown at the bottom of the figure. The most attentive person is indicated by a rectangle, and the attentiveness ranking is labeled by a number above each person.

At frame 50, person no. 1 was detected as a speaking person. His attentiveness became larger than the attentiveness of person no. 2. Examining frames 100 to 120, the proposed attention model chose person no. 2 as the most attentive person, while human evaluation considered person no. 1 as the most attentive person. The outcomes were different because both participants were located at similar distances, making it difficult for people to determine whether person no. 1 or person no. 2 was closer. Consequently, the most attentive person selection based on distance becomes critical. However, in the case of attentiveness quantification by a robot, the distance is computed as a real number, so determining the closest person among participants is an easy task.

At frames 129 and 210, the participant (person no. 1) turned his head and looked away from the robot. This resulted in a decrease in his attentiveness, and made another participant become more interesting to the robot. As a result, the other participant’s attentiveness significantly increased. Frame 250 demonstrates a possible error in the selection of the most attentive person caused by consecutive false speaking status detection.

**Figure 11:**
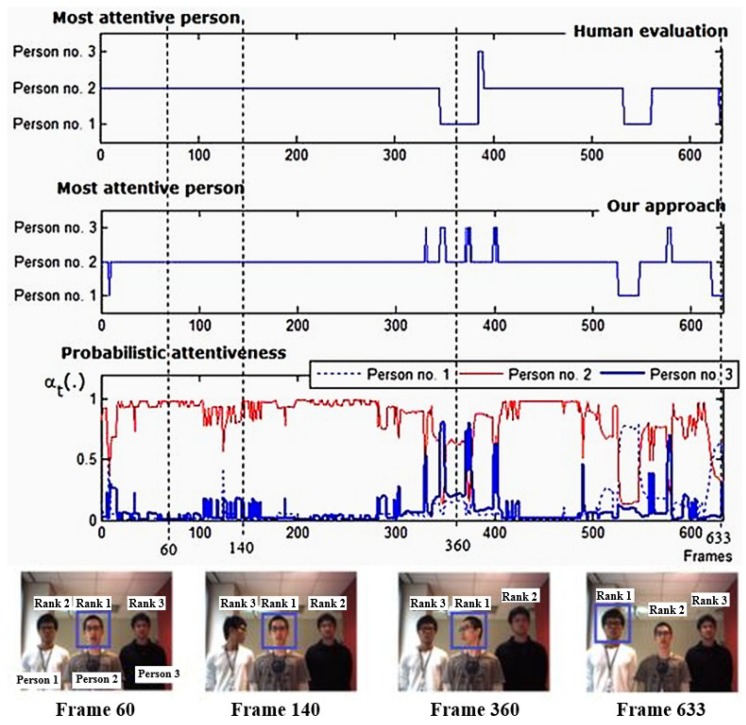
Robot’s attention results, showing a performance of our attention approach in the sense of constant in the number of participants in a robot’s view (three persons situation).

Figure 11 depicts the attention outcomes of one image sequence of a situation of three participants. The middle graph shows the selection of the most attentive person over time compared to the human evaluation, which is illustrated in the top graph. The bottom graph depicts the computed probabilistic attentiveness of participants in the robot’s view.

In frames 350–370 (Figure 11), the proposed attention approach chose person no. 2 as the most attentive person instead of person no. 1, and there were several undesired attention shifts. These were caused by continuous errors in the estimated observations. These errors were due to consecutive errors in the estimation of the head-pan angles of person no. 2. Hence, despite the effective probabilistic attentiveness computation for the most attentive person selection and people attention prioritization, our approach cannot withstand extreme error in observations if the error occurs continuously for a long period of time.

The proposed approach performed well, as expected in terms of determining the most attentive person (Figure 10 at frames 50, 129, and 210, and Figure 11 at frame 60, 140, and 663). Even when there was no speaking person present, the proposed approach was able to determine the most attentive person, such as in frames 70–300 in Figure 10 and in frames 140 and 663. In the absence of a speaking person, the transition probabilities of the robot’s FOA become equally well-distributed according to the adaptable state transition probabilities described in Section 4.1.2. As a result, the selection procedure of the most attentive person is efficiently conducted and altered by other visual features.

**Figure 12:**
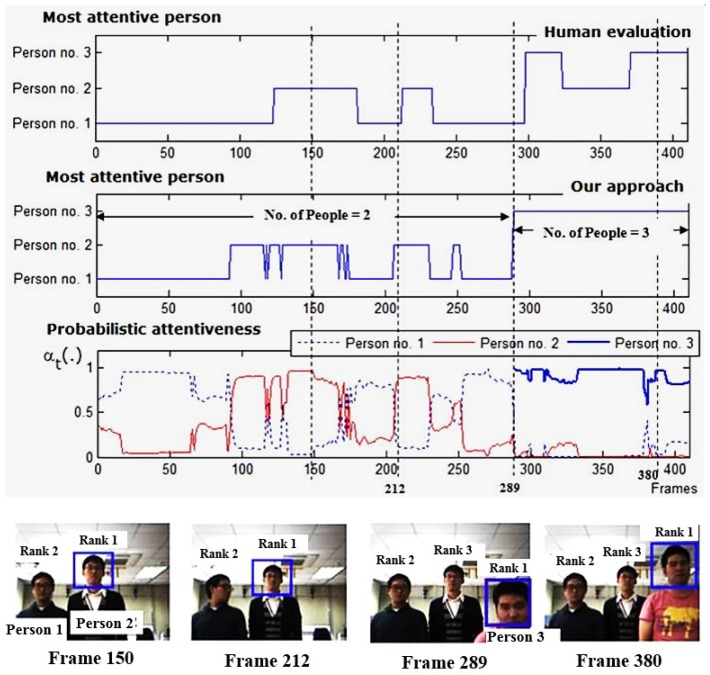
Robot’s attention results, showing a performance of our attention approach in the sense of change in the number of participants in a robot’s view (two persons to three persons situation).

Figure 12 depicts the attention outcomes of one image sequence of a situation, in which there is a change in the number of participants. At the beginning, there were two participants in the robot’s view. Our proposed attention approach prioritized these two persons with respect to the computed attentiveness. Starting from frame 289, a new person appeared and stayed in the robot’s view. The current number of people in a robot’s view became three. That person was included automatically and seamlessly into the attention model, and his attentiveness was calculated and compared with those of the other participants. This confirms the scalability of our proposed attention model based on the Scalable HMM in terms of the change in the number of people and observations. The model scalability also applies to situations with a decreasing number of participants.

The set of event conditions [38] for the determination of a robot’s FOA are listed as follows:

- If the robot detects a speaking person, the speaker becomes the most attentive person.
- As long as the person is speaking, the speaking of other people is ignored.
- When the attentive person stops talking for more than two seconds, the robot loses its anchor on that person as being the most attentive person.
- Only a speaking person can take over the role of the most attentive person.

**Figure 13:**
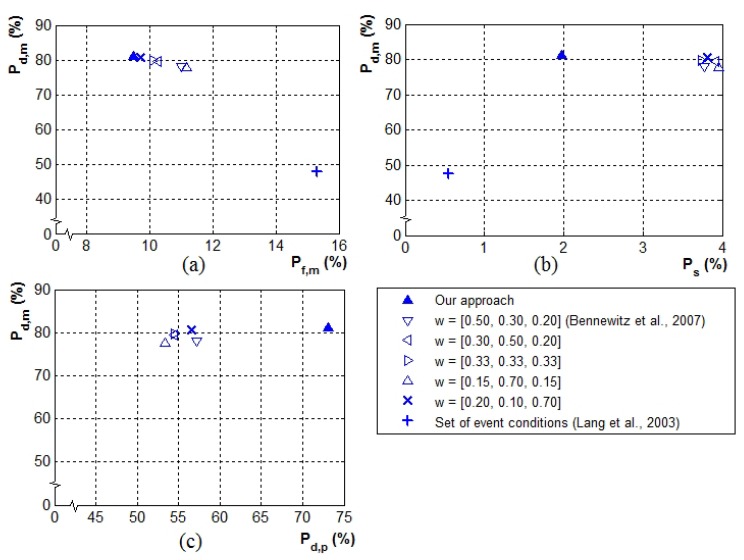
Performance comparison: (**a**,**b**) receiver operating characteristic (ROC) most attentive person detection space and plots of the proposed approach and two other attention approaches; (**c**) probability plots of the most attentive person detection $P_{d,m}$ vs. the people attention prioritization $P_{d,p}$.

For the heuristic approach, the weighted sum approach [42] is tested. Five sets of pre-defined weights (Figure 13) for the three ostensive-stimuli were investigated to explore the approach’s performance, where $w=[w_{d},w_{h},w_{s}]$ is a set of weights, and $w_{d}$, $w_{h}$, and $w_{s}$ are weights for distance, head-pan angle, and speaking status, respectively.

The performance comparison in terms of the most attentive person selection using the receiver operating characteristic (ROC) space is shown in Figure 13a,b. The ROC curve is a graphical plot which illustrates the performance of a system via the comparison of two relative operating characteristics [52,53]. Figure 13c shows a performance comparison with respect to both the most attentive person selection and people attention prioritization. The plots show that our proposed robot attention model outperforms these two heuristic attention approaches.

Our attention model succeeded in obtaining a high detection rate of the most attentive person (≈76% of $P_{d,m}$) and the highest detection rate of people attention prioritization (≈75% of $P_{d,p}$) compared to the other two approaches. The proposed approach also achieved a small rate of attention shift during intervals, $P_{s}$, which was only (≈2%). Additionally, $P_{d,m}$ was improved by over 30% compared to Lang et al.’s approach (the approach using the set of event conditions) and by almost 3% compared to Bennewitz et al.’s weighted sum approach. Compared to the other two attention approaches, the proposed approach had significant improvement of almost 20% regarding $P_{d,p}$ and approximately 2% regarding $P_{s}$.

For the approach using the set of event conditions [38], although it achieved a very small rate of $P_{s}$ (≈1%), an extremely low rate of $P_{d,m}$ (≈47%) and a rather high rate of $P_{f,m}$ (≈16%) resulted. $P_{d,p}$ could not be calculated in this case because the designed event conditions were too simplified and did not cover the issue of people attention prioritization.

Considering the performance of the weighted sum approach [42], for all five weight sets, there were similar performances despite the differences in weight sets (≈73% of $P_{d,m}$, ≈54 of $P_{d,p}$, 14% of $P_{f,m}$, and 4% of $P_{s}$). This indicates that a fixed weight distribution of stimuli did not guarantee the optimum performance of the attention model. The approach with one fixed weight set might result in a high detection rate of the most attentive person, but may deliver a low detection rate of people attention prioritization, with high susceptibility to frequent undesired attention shifts, or vice versa. This implies that with the heuristic equation approach, the determination of suitable parameters that ensure the optimum trade-off between the hit rate and false alarm rate is critical.

**Figure 14:**
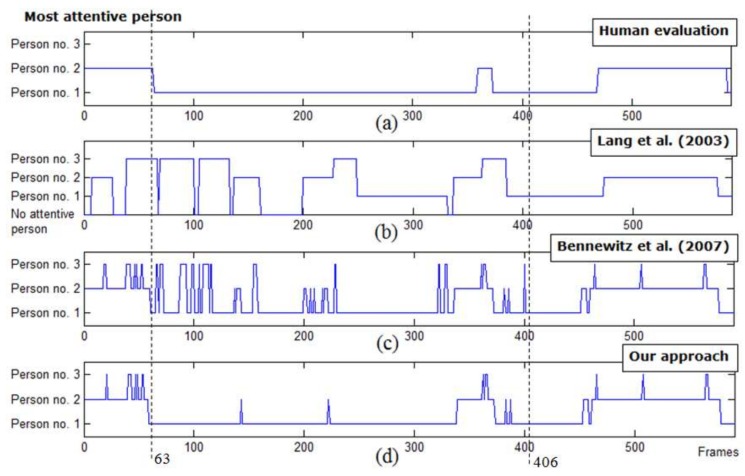
The most attentive person selection comparison between our attention approach and two other approaches, showing some false detection intervals of the most attentive person and improvements in terms of undesired attention shift moderation.

Figure 14 depicts the most attentive person selection outcomes of Lang et al.’s approach (the set of event conditions), Bennewitz et al.’s approach (the weighted sum approach), and our proposed approach tested on one image sequence of the three-person situation, compared to human evaluation.

The first graph (Figure 14a) illustrates human evaluation of the most attentive person. The second graph (Figure 14b) depicts the result of Lang et. al.’s approach. Note that, compared to Figure 14c,d, there were several noticeable undetermined intervals of the most attentive person in Figure 14b (i.e., losing attention from the most attentive person). Specifically, these occurred during intervals in which no speaking person was detected. As a result, the attentive person was not unable to be determined by the proposed method.

The third graph (Figure 14c) depicts the result of Bennewitz et al.’s approach ($w=[w_{d}=\;0.20,$$w_{h}=0.10,w_{s}=0.70]$). Next, the fourth graph (Figure 14d) shows the result of our proposed method. Considering frames 63–406 in Figure 14b–d, as expected from the probabilistic approach, several undesired attention shifts were moderated while correct detections of the most attentive person were maintained.

## 7. Conclusions

A novel vision-based attentiveness determination method has been presented to improve a robot attention model’s performance in determining the most attentive person and prioritizing people based on attentiveness. Additionally, the effective computation of attentiveness and adaptation to changes in the number of participants and observations was accounted for in the proposed method. The proposed approach is based on relevance theory, a human communication methodology that explains how people evaluate the attention of other people during interactions.

The proposed approach consists of a computation method for probabilistic stimuli-relevance and Scalable HMM, an attentiveness determination model for most attentive person selection and people attention prioritization. Unlike the conventional HMM, the Scalable HMM has a scalable number of states and observations, and online adaptability of the state transition probabilities with respect to changes in the number of states. Furthermore, for effective attentiveness determination, the speaking status of people was employed as conditional parameter for adaptable state transition probabilities, unlike most previous attention approaches. A better, more robust attentiveness determination was achieved, wherein the selection of the most attentive person could be conducted even in situations where no speaking person was detected. By employing online forward analysis, the probabilistic attentiveness of each person can be determined in real-time with low computation cost. A comparison of the computed attentiveness of people yielded the most attentive person selection and the people prioritization based on their attentiveness. The parameters of our proposed approach can be efficiently and conveniently learned based on PSO, such that good resistance to noisy observations and a good performance rate was achieved. The approach was successfully tested on 10 image sequences (7567 frames) with encouraging experimental results (≈76% accuracy in most attentive person detection and more than 75% accuracy for people attention prioritization). Additionally, the proposed method works robustly online in various lighting conditions and with changes in the number of participants. Compared to the two other more conventional attention approaches, improvements of nearly 20% in people attention prioritization and 2% in resisting undesired attention shifts were achieved. Overall, the most optimal performance was presented with the proposed method.

Despite being sufficiently robust in lighting variation, low-resolution images, and noisy observations, the proposed vision-based attentiveness determination for the most attentive person selection and people attention prioritization cannot operate well under extremely poor lighting conditions; in such conditions, image noise is high, resulting in extreme error in observations and a poor performance rate with the proposed model. Hence, in order to improve the model’s efficiency, additional ostensive-stimuli could be included into the attentiveness determination model, such as hand gestures, facial expression, and natural language that can be trained using various deep learning architectures in [54,55]. Furthermore, to succeed in imitating a more human-like the attention system, a robot’s head-eye control system and audio information could also be incorporated with visual information. In this manner, the robot could consider both vision and sound for its decision-making process, as humans do, thus improving its human likeness.

## Appendix A. Vision-Based Detection Methods for Distance of Person-to-Robot, Head-Orientation, and Speaking Statuses

In this section, vision-based detection approaches for three attention-related visual features (i.e., the distance of person-to-robot, head-orientation, and speaking statuses of a person) which are commonly observed and employed in the robot’s attention model, are briefly described.

### Appendix A.1. Distance of Person-to-Robot

Referring to psychological studies in personal space zones [56,57], humans regard their surrounding region (personal space) such that any invasion of personal space is always brought into attention. Table A1 summarizes the personal space zones between humans, so-called Hall’s distances.

**Table A1:** Human–human personal space zones (Hall’s distances).

Personal Space Zone	Range	Situation
Close Intimate Zone	0 m. to 0.15 m.	Lover of close friend touching
Intimate Zone	0.15 m. to 0.45 m.	Lover of close friend only
Personal Zone	0.45 m. to 1.2 m.	Conversation between friends
Social Zone	1.2 m. to 3.6 m.	Conversation to non-friends
Public Zone	over 3.6 m.	Public speech making

Walters et al. [58] presented a study in human–robot social space zones. The study shows that the human–robot interpersonal distances are comparable to those found in human–human interpersonal distances (see Table A1). Thus, the concept of Hall’s distances can also apply to robots.

Here, to acknowledge people’s whereabouts in a robot’s view, we visually detect a person-to-robot distance using a BumbleBee2 stereo camera. By employing a stereo camera, a disparity image is computed and is readily accessible in real-time. To estimate a person-to-robot distance, first, a person’s face should be located in the image. This can be done by a face detector algorithm. The face detector used in our system is described in several studies [59,60,61]. This face detector is one of the most powerful and widely used systems described in, and proposed by, the literature. Next, by averaging the intensity values of all existing pixels inside the located face window in the disparity image, the person-to-robot distance can be effectively estimated.

### Appendix A.2. Head Orientation

In general, during an interaction between humans, the gaze direction is an important factor which notifies the object/subject of interest (the robot) of a gazer’s interest (i.e., that of a human) at a particular time. Typically, the gaze can be determined from a combination of two basic actions: head-orientation and eyeball-motion.

Particularly, for accurate eyeball-motion detection and tracking, an adequate—or even high-resolution—face image sequence is necessary. However, as is known in MPRI, humans should be far enough away such that they can all fit in the robot’s view. As a result, high-resolution face image sequences become a luxury difficult to obtain. Unavoidably, this raises difficulties in eyeball-motion detection and tracking. Hence, for simplicity in improving the accuracy with which a gaze is detected accuracy, we operate under the assumption that a human’s looking-direction is the same direction as a human’s facing-direction. In this way, the head orientation simply becomes a representation of the gaze direction.

Specifically, a structure of human’s head has three degrees of freedom (3 DOF), with one rotational DOF around the neck (head pan) and two DOF at the neck (i.e., head tilt: looking up/down and tilting head left/right). Furthermore, to make the estimation less complex, this study considers only the head pan as a respective head movement for attracting a robot’s attention. Therefore, in this case, the other DOFs at the neck (i.e., head tilt vertically and horizontally) are ignored and treated as noise motions.

To estimate the person’s head pan, a coarse head-pose estimation algorithm is considered in this study. This approach can still efficiently estimate the person’s head orientation in low-resolution face images. Brown and Tian explored the merits of two robust coarse head-pose estimation approaches [62].

To choose the applicable approach for our application, we tested the two approaches on the same dataset and found that the NN model approach was more robust and reliable than the probabilistic model approach. Hence, in this study, the NN-based coarse head pose estimation, similar to Zhao et al. [63], is employed to estimate a person’s head pan angle.

### Appendix A.3. Speaking Statuses of a Person

An act of speaking (i.e., calling a name, talking, shouting, etc.) is commonly considered an influential factor involved in attracting another person’s attention. Two significant speaking statuses are concerned in this paper: speaking and non-speaking statuses.

Particularly, the speaking status detection approach should efficiently work online with low-resolution mouth images and in various lighting conditions because, in the case of MPRI, the participants should be located far enough away from the robot such that they can be in the robot’s narrow view. Furthermore, the approach should be robust enough to withstand frequent undesired speaking state changes, which are often caused by noisy observation.

To overcome these requirements, a probabilistic approach described in Tiawongsombat et al. [25] is employed herein. The approach is a bi-level HMM, which analyzes lip activity energy on a mouth image to detect speaking statuses of a person in real-time. Specifically, the speaking status detection method is based on lip movement and speaking assumptions, thus embracing two essential consecutive procedures (post-processing and classification procedures) into a single model. Further, a bi-level HMM is an HMM with two state variables in different levels, where state occurrence in a lower level conditionally depends on the state in an upper level. Refer to [25] for more details.
